# Pigmented vulvar basal cell carcinoma in a Hispanic patient treated with Mohs micrographic surgery

**DOI:** 10.1097/JW9.0000000000000121

**Published:** 2023-12-06

**Authors:** McKayla Poppens, Katrina Lee, Michael O. Nguyen

**Affiliations:** a David Geffen School of Medicine, University of California Los Angeles, Los Angeles, California; b Division of Dermatology, Department of Medicine, David Geffen School of Medicine, University of California Los Angeles, Los Angeles, California

**Keywords:** labial neoplasm, Mohs micrographic surgery, pigmented vulvar basal cell carcinoma, vulvar neoplasm

**What is known about this subject in regard to women and their families?**

- Pigmented basal cell carcinoma is a rare cause of vulvar neoplasms that has previously been treated with wide local excision or vulvectomy.

**What is new from this article as messages for women and their families?**

- This article highlights the importance of genital examination in skin examinations, describes the presentation and mimickers of vulvar-pigmented basal cell carcinoma, and most importantly, encourages the use of Mohs micrographic surgery for vulvar pathologies to minimize disfigurement, as well as psychological and sexual consequences of other surgical approaches.

## Dear Editors,

### Case presentation

A 52-year-old Hispanic female with a history of undifferentiated connective tissue disease and autoimmune hepatitis presented to a dermatology clinic for a bleeding pigmented lesion on her labia majoris. The patient initially noted the growth 3 years prior, when it bled for several days. She denied a history of preceding trauma, radiation, or surgical procedures in the area. Past cervical screening tests were negative for HIV and high-risk human papillomavirus subtypes. She had no personal or family history of skin cancer. 11 months before presentation, she began azathioprine for autoimmune hepatitis.

**Fig. 1.**
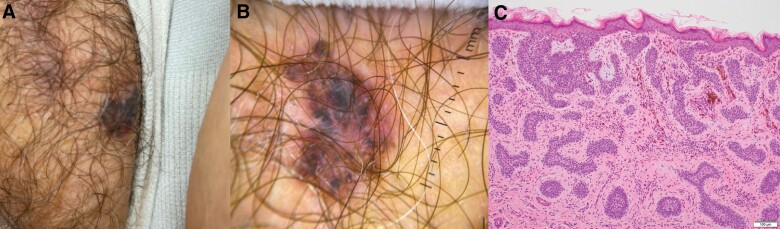
(A) Pigmented plaque on the right labia majoris. (B) Dermoscopy revealed a 1.4 cm irregularly shaped plaque with brown-black leaf-like structures and hazy red and white structureless areas. (C) Basaloid nodules and micronodules with peripheral palisading of cells invading the dermis. Melanin pigment is seen within the tumor deposits (hematoxylin-eosin, 10×).

Physical examination revealed a 1.4 cm irregularly shaped, inflamed plaque with black pigmentation on the right labia majora (Fig. 1A). The surrounding skin was without signs of photodamage. Under dermoscopy, black leaf-like structures and hazy red and white structureless areas were identified (Fig. 1B). Histopathology revealed characteristic basaloid islands and pigment incontinence consistent with pigmented, nodular, and micronodular basal cell carcinoma (BCC) (Fig. 1C). The tumor was treated with Mohs micrographic surgery (MMS), and there were no signs of recurrence at 4-month follow-up.

### Discussion

BCC is the most common form of skin cancer. However, vulvar BCC is rare, accounting for less than 5% of vulvar neoplasms.^1^ The prevalence of pigmented vulvar BCC is unknown. To our knowledge, this is the first reported case of a pigmented vulvar BCC in an immunosuppressed Hispanic patient treated with MMS.

A total of 27 cases of pigmented vulvar BCCs have been described between 2005 and 2023. Pigmented vulvar BCC affects a wide range of ages (30–87 years old) and often presents with a delay of several months before patients seek care (2 months–20 years). On evaluation, the pigmented lesions vary in size from 2.5 mm to 6 cm (2.13 cm average). Approximately 66% of patients (18/27) were of Asian ethnicity (Table 1).^2-10^

**Table 1:** Clinical characteristics of patients with pigmented vulvar basal cell carcinoma

Authors	No. of patients	Age	Ethnicity	Gross morphology/symptoms	Size (cm)	Histologic subtype	Invasion	Delay to presentation (mo)	Treatment	Length of recurrence-free follow-up (y)	Potential risk factors identified
de Giorgi *et. al.* (2005); Italy	2	Unk.	Unk.	Unk.	1–5	Unk.	No	Unk.	Unk.	Unk.	No
Lui *et. al.* (2009); China	1	75	Asian	Pigmented nodule, ulcerated, painful	2	Mixed (superficial and nodular)	No	Unk.	Wide local excision	10	No
	1	59	Asian	Pigmented papule	0.3	Nodular	No	Unk.	Excision	2	No
	1	81	Asian	Pigmented nodule	1.8	Mixed (superficial and nodular)	No	Unk.	Excision	7	Vaginal intraepithelial neoplasia grade I
	1	72	Asian	Pigmented papule	0.6	Mixed (superficial and nodular)	No	Unk.	Wide local excision	7	No
	1	69	Asian	Pigmented papule, painful	0.5	Nodular	No	Unk.	Excision	7	No
	1	83	Asian	Pigmented nodule	1.3	Nodular	No	Unk.	Excision	12	No
	1	81	Asian	Pigmented nodule, ulcerated	2	Nodular	No	Unk.	Excision	1	No
	1	73	Asian	Pigmented nodule, ulcerated	3	Mixed (superficial and nodular)	No	Unk.	Excision	1	Cervical squamous cell carcinoma with radiotherapy, >10 y prior
	1	78	Asian	Pigmented nodule, ulcerated	3	Nodular	No	Unk.	Excision	2	No
	1	84	Asian	Pigmented nodule	3	Nodular	No	Unk.	Excision	8	No
	1	82	Asian	Pigmented fungating tumor with satellite nodules, ulcerated, bleeding	6	Mixed (nodular and infiltrative)	Perineural invasion	Unk.	Radical vulvectomy, bilateral lymphadenectomy	0.5	No
	1	86	Asian	Pigmented nodule	6	Nodular	No	Unk.	Excision	0.17	No
	1	90	Asian	Pigmented nodule	1.5	Nodular	No	Unk.	Bilateral simple vulvectomy	0.25	No
Kanitakis *et. al.* (2011); France	1	87	Caucasian	Erythematous, infiltrated plaque with hyperpigmented border, pruritic	Unspecified	Nodular	No	Unk.	N/A, lost to follow-up	N/A	No
Yaghoobi *et. al.* (2011); Iran	1	78	Caucasian	Pigmented patch	4	Unk.	No	240	Wide local excision, lymph node dissection	Unk.	No
Mulvany et. al. (2012); Australia	1	74	Unk.	Cream plaque with erythematous margin, pruritic	0.8	Mixed (infiltrative, superficial, multinodular)	No	9	Excision	4	Perianal squamous cell carcinoma, 6 y prior; Lichen sclerosis
Namuduri *et. al.* (2019); China	1	67	Asian	Pigmented nodule	1	Nodular	No	6	Wide local excision	Unk.	No
	1	73	Asian	Pigmented nodule	1	Multifocal, superficial	No	7	Wide local excision	Unk.	No
	1	56	Asian	Pigmented papule, pruritic, bleeding	2	Nodular	No	6	Simple vulvectomy	Unk.	No
	1	85	Asian	Pigmented nodule	1.5	Nodular	No	6	Right hemivulvectomy	Unk.	Lichen Sclerosis
	1	30	Asian	Pigmented nodule	4	Mixed (nodular, with squamous differentiation)	No	2	Radical vulvectomy, lymph node dissection	Unk.	No
Bertaina *et. al.* (2019); Argentina	1	73	Unk.	Pigmented nodule, central ulceration	0.25	Mixed (nodular, infiltrative)	No	Unk.	Excision	Unk.	No
Zapecova *et. al.* (2021); Czech Republic	1	85	Unk.	Unk.	Unspecified	Unk.	Unk.	Unk.	Unk.	Unk.	Unk.
Camela *et. al.* (2022); Italy	1	77	Caucasian	Indurated plaque, ulcerated	Unspecified, appears multi-cm per photo	Mixed (infiltrative, basosquamous, morpheaform)	Yes, unspecified	240	Sonidegib, 10 months	10	No
Current case (2023); USA	1	52	Hispanic	Pigmented macule, bleeding	1.4	Mixed (nodular, micronodular)	No	36	Mohs micrographic surgery	4	No

N/A, not applicable; Unk., unknown.

Vulvar BCC can sometimes be an aggressive neoplasm with deep local extension and perineural invasion.^11^ Two out of 27 (7.4%) pigmented vulvar BCC cases reported either perineural or perivascular invasion and were at least 6 cm at presentation. Conventionally, vulvar BCCs have been treated with local excision or vulvectomy, which have higher risks of disfigurement, and psychological and sexual consequences.^12^ Moreover, the risk of recurrence with local excision is 20%, and metastases have been reported.^13^ Our patient is the first pigmented vulvar BCC case reported in the literature that has been treated with MMS, an efficacious method for all vulvar malignancies.^12^ No recurrences of vulvar BCC have been reported with MMS.^12^

In summary, we present a case of a pigmented BCC on the vulva of an immunosuppressed Hispanic patient following a 3-year delay in diagnosis, which was treated with MMS, and review the current literature of other cases. Given the atypical location and presentation, pigmented vulvar BCCs can be a diagnostic challenge. We encourage clinicians to consider pigmented BCC on their differential for atypical pigmented vulvar neoplasms. Importantly, skin cancer screenings should include thorough genital examination in all populations, especially in immunosuppressed patients. Additional studies are necessary to clarify the pathogenesis and optimal management of pigmented vulvar BCC.

## Conflicts of interest

None.

## Funding

None.

## Study approval

N/A

## Author contributions

MP: Performed the literature review and drafted the manuscript. KL and MON: Critically revised the manuscript.

## Patient consent

Consent for publication of the patient photographs was obtained at the time of article submission. A copy of the submission was made available for the patient.

## References

[1] Mulayim N Foster Silver D Tolgay Ocal I Babalola E. Vulvar basal cell carcinoma: two unusual presentations and review of the literature. Gynecol Oncol 2002;85(3):532–7. doi: 10.1006/gyno.2001.6582. 12051887 10.1006/gyno.2001.6582

[2] de Giorgi V Salvini C Massi D Raspollini MR Carli P. Vulvar basal cell carcinoma: retrospective study and review of literature. Gynecol Oncol 2005;97(1):192–4. doi: 10.1016/j.ygyno.2004.12.008. 15790457 10.1016/j.ygyno.2004.12.008

[3] Lui PC Fan YS Lau PP. Vulvar basal cell carcinoma in China: a 13-year review. Am J Obstet Gynecol 2009;200(5):514.e1–5. doi: 10.1016/j.ajog.2008.12.004. 10.1016/j.ajog.2008.12.004 19200934

[4] Kanitakis J Arbona-Vidal E Faure M. Extensive pigmented vulvar basal-cell carcinoma presenting as pruritus in an elderly woman. Dermatol Online J 2011;17(1):8. 21272499

[5] Yaghoobi R Razi T Feily A. Clinical image: an unusual pigmented basal cell carcinoma arising from vulva. Acta Dermatovenerol Alp Pannonica Adriat 2011;20(2):81–2. 21993706

[6] Mulvany NJ Rayoo M Allen DG. Basal cell carcinoma of the vulva: a case series. Pathology 2012;44(6):528–33. doi: 10.1097/PAT.0b013e328357a001. 22935972 10.1097/PAT.0b013e328357a001

[7] Namuduri RP Lim TY Yam PK. Vulvar basal cell carcinoma: clinical features and treatment outcomes from a tertiary care centre. Singapore Med J 2019;60(9):479–82. doi: 10.11622/smedj.2019014. 30773599 10.11622/smedj.2019014 PMC7911082

[8] Bertaina C Salerni G Celoria M. Dermoscopy of pigmented vulvar basal cell carcinoma. Dermatol Pract Concept 2019;9(3):239–40. doi: 10.5826/dpc.0903a20. 31384508 10.5826/dpc.0903a20 PMC6659589

[9] Zápecová K. Pigmented vulvar lesions - review and case report focusing on pigmented basal cell carcinoma. Ceska Gynekol 2021;86(5):325–30. doi: 10.48095/cccg2021325. 34736330 10.48095/cccg2021325

[10] Camela E Villani A Scalvenzi M Costa C. Giant basal cell carcinoma of the vulva successfully treated with Sonidegib. Dermatol Ther 2022;35(9):e15723. doi: 10.1111/dth.15723. 35851517 10.1111/dth.15723 PMC9540618

[11] Miller ES Fairley JA Neuburg M. Vulvar basal cell carcinoma. Dermatol Surg 1997;23(3):207–9. doi: 10.1111/j.1524-4725.1997.tb00025.x. 9145965 10.1111/j.1524-4725.1997.tb00025.x

[12] Shwe S Elsensohn AN Ortiz C Kraus CN. Mohs micrographic surgery for vulvar malignancies: a systematic review. J Am Acad Dermatol 2022;87(1):159–62. doi: 10.1016/j.jaad.2021.06.875. 34237353 10.1016/j.jaad.2021.06.875

[13] Winkelmann SE Llorens AS. Metastatic basal cell carcinoma of the vulva. Gynecol Oncol 1990;38(1):138–40. doi:10.1016/0090-8258(90)90027-i. 2191906 10.1016/0090-8258(90)90027-i

